# Disease progression in Sanfilippo type B: Case series of Brazilian patients

**DOI:** 10.1590/1678-4685-GMB-2023-0285

**Published:** 2024-03-08

**Authors:** Yorran Hardman Araújo Montenegro, Francyne Kubaski, Franciele Barbosa Trapp, Mariluce Riegel-Giugliani, Carolina Fischinger Moura de Souza, Erlane Marques Ribeiro, Charles Marques Lourenço, Augusto César Cardoso-dos-Santos, Márcia Gonçalves Ribeiro, Chong Ae Kim, Matheus Augusto Araújo Castro, Emília Katiane Embiruçu, Carlos Eduardo Steiner, Filippo Pinto e Vairo, Guilherme Baldo, Roberto Giugliani, Fabiano de Oliveira Poswar

**Affiliations:** 1 Hospital de Clínicas de Porto Alegre, Serviço de Genética Médica, Porto Alegre, RS, Brazil.; 2 Universidade Federal do Rio Grande do Sul, Instituto Nacional de Genética Médica Papulacional, Porto Alegre, RS, Brazil.; 3 Hospital de Clínicas de Porto Alegre, Serviço de Genética Médica, Rede MPS Brasil, Porto Alegre, RS, Brazil.; 4 Casa dos Raros, Porto Alegre, RS, Brazil.; 5 Hospital Infantil Albert Sabin, Serviço de Genética Médica, Fortaleza, CE, Brazil.; 6 Centro Universitário Estácio, Ribeirão Preto, SP, Brazil.; 7 Universidade Federal do Rio de Janeiro, Instituto de Puericultura e Pediatria Martagão Gesteira, Serviço de Genética Médica, Rio de Janeiro, RJ, Brazil.; 8 Universidade de São Paulo, Faculdade de Medicina, Hospital das Clínicas, Instituto da Criança, São Paulo, SP, Brazil.; 9 Universidade Federal da Bahia, Complexo Hospitalar Universitário Professor Edgar Santos, Departamento de Ciências da Vida, Salvador, BA, Brazil.; 10 Universidade Estadual de Campinas, Faculdade de Ciências Médicas, Departamento de Medicina Translacional, São Paulo, SP, Brazil.; 11 Universidade Federal do Rio Grande do Sul, Departamento de Fisiologia, Porto Alegre, RS, Brazil.; 12 Dasa Genômica, São Paulo, SP, Brazil; 13 Mayo Clinic, Center for Individualized Medicine, Rochester, MN, USA.; 14 Mayo Clinic, Department of Clinical Genomics, Rochester, MN, USA.; 15 Universidade Federal do Rio Grande do Sul, Programa de Pós-Graduação em Genética e Biologia Molecular, Porto Alegre, RS, Brazil.

**Keywords:** Sanfilippo syndrome, Mucopolysaccharidosis IIIB, MPS Brazil Network, lysosomal storage diseases, heparan sulfate, Brazil

## Abstract

Mucopolysaccharidosis type IIIB (MPS IIIB) is caused by deficiency of alpha-N-acetylglucosaminidase, leading to storage of heparan sulphate. The disease is characterized by intellectual disability and hyperactivity, among other neurological and somatic features. Here we studied retrospective data from a total of 19 MPS IIIB patients from Brazil, aiming to evaluate disease progression. Mean age at diagnosis was 7.2 years. Speech delay was one of the first symptoms to be identified, around 2-3 years of age. Behavioral alterations include hyperactivity and aggressiveness, starting around age four. By the end of the first decade, patients lost acquired abilities such as speech and ability to walk. Furthermore, as disease progresses, respiratory, cardiovascular and joint abnormalities were found in more than 50% of the patients, along with organomegaly. Most common cause of death was respiratory problems. The disease progression was characterized in multiple systems, and hopefully these data will help the design of appropriate clinical trials and clinical management guidelines.

Mucopolysaccharidosis (MPS) is a group of lysosomal storage disorders resulting from deficiency of enzymes involved in the degradation of glycosaminoglycans (GAGs). MPS IIIB is caused by deficiency of alpha-N-acetylglucosaminidase (NAGLU, E.C. 3.2.1.50, OMIM 252920) (Neufeld and Muenzer, 2001) associated with biallelic variants in the *NAGLU* gene. To date, no clear correlation has been observed between variants in the *NAGLU* gene and the clinical presentation of the pathology, since a series of factors can contribute to the heterogeneity of the disease (Montenegro et al., 2022). It is possible to classify MPS IIIB patients based on the clinical presentation and the severity of their symptoms as severe or attenuated. However, these classifications do not concern the variant-symptomatology correlation, rather, only the clinical presentation (Valstar et al., 2011). The main characteristic of MPS III is intellectual disability and hyperactivity due to progressive neurodegeneration. 

The age of onset and progression of the disease is variable, but three stages are reported (Cross et al., 2014; Kim et al., 2016): i) in the first years of life, developmental delay appears after an initial normal phase; ii) progressive mental deterioration and behavioral problems begins around 3 to 4 years of age; and, iii) motor difficulties, swallowing problem and spasticity appear, while the behavioral alterations tend to disappear. Patients usually die by the second or third decade of life (Valstar et al., 2011).

The progression of MPS IIIB has been recently reported by groups from North America, Europe and Asia, but there is limited data on South American patients (Whitley et al., 2018; Lin et al., 2019). In the present study, we gathered information of 19 (sixteen unrelated) Brazilian MPS IIIB patients from different centers. We assessed clinical and biochemical information of these patients to analyze disease progression.

A retrospective study was carried out for patients diagnosed with MPS IIIB born from 1989 to 2016 and followed in the several medical Brazilian centers: Universidade Estadual de Campinas (Campinas, São Paulo), Hospital Universitário Professor Edgard Santos (HUPES) (Salvador, Bahia), Hospital Infantil Albert Sabin (Fortaleza, Ceará), Centro Universitário Estácio de Ribeirão Preto (Ribeirão Preto, São Paulo), Hospital de Clínicas de Porto Alegre (Porto Alegre, Rio Grande do Sul), and Instituto da Criança do Hospital das Clínicas da FMUSP (São Paulo, São Paulo). To be included in the present communication, patients had to have biochemical (NAGLU activity) and/or mutational analysis confirming NAGLU deficiency. Patients’ charts were reviewed for biochemical findings, medical history, clinical manifestations and assessments. Any available results of the following investigations were also recorded: electroencephalography (EEG); electrocardiography (ECG); echocardiography; hearing assessment by pure-tone audiometry; tympanometry; physical exam; polysomnography, as well as other information relevant to the course of disease, including surgical procedures and use of medication. The MPS Brazil Network provided the data, and it is a project approved by the ethics committee from Hospital de Clínicas de Porto Alegre (GPPG # 03-066). Written informed consent was acquired from a parent for children or from patients over 18 years, allowing the use of images.

The present study assessed retrospective data of MPS IIIB patients diagnosed and followed in expert reference centers in Brazil. Age at diagnosis was 7.2 years (Figure 1 a ), younger than the average time previously reported for patients from the whole country (Montenegro et al., 2022) and closer to findings from Taiwan, USA, UK and Germany (Whitley et al., 2018; Lin et al., 2019).

**Figure 1 -:**
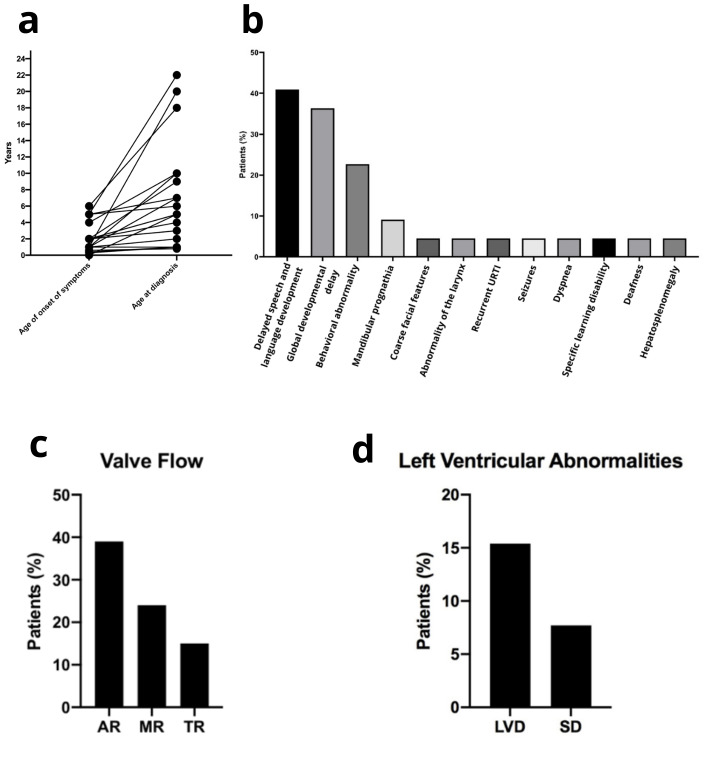
Findings in MPS IIIB patients. a. Relationship between age at onset of symptoms and age at diagnosis (n=12). The data reveals a diagnostic delay in Brazil. b. Presenting signs and symptoms at diagnosis (n=22). Notice the lack of significant somatic involvement in a majority of patients before diagnosis. c and d. Cardiac findings detectable on echocardiogram (n=13) related to valve flow (c) and left ventricular dimensions and contractibility during follow-up of patients (d). Notice how heart abnormalities become frequent later in life. Other somatic alteration can be found in the Table S1. AR, aortic regurgitation; LVD, Left ventricle dilatation; MR, mitral regurgitation; SD, systolic dysfunction; TR, tricuspid regurgitation; URTI, upper respiratory tract infection.

We were able to obtain clinical records for 19 patients from birth to their last visit to the physician, including 16 unrelated patients. We firstly looked for possible neonatal findings. A percentage of 54.5% of births were by cesarean delivery and 45.5% of births by vaginal delivery. Newborns had an average weight of 3,154 g (IQR 2,862g - 3,550g) (n=22), with an average length of 48.7 cm (IQR 46.8 cm - 50.3 cm) (n=16) and cephalic perimeter of 34.5 cm (IQR 33.87 cm - 35 cm) (n=8). One patient showed extensive Mongolian spots at birth. Other neonatal conditions reported in a single patient were: jaundice after 48 hours, oligohydramnios, coarse facies, umbilical hernia, neonatal asphyxia, nuchal cord requiring hospitalization, hypertrichosis, synophrys, and hepatosplenomegaly. A summary can be found in Table 1.

**Table 1 -:** Clinical and laboratory data on Brazilian MPS IIIB patients.

No	Gender	Current age (Yrs)	Age at diagnosis (Yrs)	NAGLU activity (reference)	Urinary GAG (ref ug/mg creatinine)	Variants	Age at onset of symptoms (yrs)	Initial symptoms	Age at first seizure (year)	Behavioral problems (age)	Cause of death (age)
1	F	7	3	0.05 (10-34)	-	-	2	Coarse facial features and Global developmental delay	NR	Sleepwalking, night terror, agitation, aggression.	Alive
2	F	5	3	0.31 (10-34)	-	NC_000017.11: c.830_832delGCT / c.830_832delGCT	NR	NR	NR	Agitation and Aggressiveness	Alive
3	F	4	0.8	0.5 (10-34)	-	-	0.6	Gingival overgrowth	NR	NR	Alive
4	F	12	10	0.12 (10-34)	226 (26-97)	NC_000017.11: c.830_832delGCT / c.830_832delGCT	1	Developmental Delay	NR	Agitation, Anxiety, Aggressiveness and Insomnia	Alive
5	M	5	1	0.00 (10-34)	505 (79-256)	NC_000017.11: c.700C>T (NP_000254.2: p.Arg234Cys) / c.700C>T (NP_000254.2: p.Arg234Cys)	0.3	Macrocephaly	NR	Irritability, Aggressiveness, Poor visual interaction	Alive
6	M	18	4	0.27 (6.6-19)	600 (67-124)	NC_000017.11: c.607C>T (NP_000254.2: p.Arg203Ter) / c.607C>T (NP_000254.2: p.Arg203Ter)	2	Delayed speech and language development	NR	NR	Alive
						NC_000017.11: c.82G>A (NP_000254.2: p.Glu28Lys) / c.82G>A (NP_000254.2: p.Glu28Lys)					
7	M	12	9	0.5 (10-34)	176 (26-97)	NC_000017.11: c.607C>T (NP_000254.2: p.Arg203Ter) / c.607C>T (NP_000254.2: p.Arg203Ter)	2	Macrocephaly	NR	Aggressiveness, Irritability	Alive
8	M	7	1	0.35 (10-34)	527 (133-274)	-	1	Upper Airway Infection	NR	Apathy	Alive
9	F	22	18	0.19 (10-34)	77 (13-45)	-	6	Agitation	18	Agitation (6) and Aggressiveness	Alive
10	F	5	3	0.00 (10-34)	123 (64-127)	NC_000017.11: c.1597C>T (NP_000254.2: p.Arg533*) / c.1597C>T (NP_000254.2: p.Arg533*)	2	Delayed speech and language development	NR	Hyperactivity (3)	Alive
						NC_000017.11: c.1811C>T (NP_000254.2: p.Pro604Leu) / c.1811C>T (NP_000254.2: p.Pro604Leu)					
11	M	7	5	0.3 (>1.5)	-	-	2	Global developmental delay	NR	NR	Alive
12	M	Deceased	20	NR^a^	-	NC_000017.11: c.222_247del (NP_000254.2: p.Val75Glyfs*108) / c.222_247del (NP_000254.2: p.Val75Glyfs*108)	1	Failure to thrive, Hepatomegaly and Diarrhea	NR	Aggresiveness (12)	Pneumonia (21)
13	F	Deceased	10	0.05 (10-34)	192 (26-97)	-	4	Global developmental delay	NR	Agitation (10)	Pneumonia (13)
14	M	Deceased	6	0.00 (10-34)	263 (26-97)	-	5	Agitation	9	Agitation	Pneumonia (13)
15	F	Deceased	22	0.12 (11-37)	124 (13-45)	-	5	Delayed speech and language development, Ataxia and Neurological Regression	NR	Hyperactivity (5)	Cardiorespiratory arrest (28)
16	M	19	5	0.00 (10-34)	340 (53-115)	-	2	Delayed speech and language development	NR	Agitation (5)	Alive
17	F	18	7	0.3 (11-37)	164 (67-124)	-	5	Delayed speech and language development and Hyperactivity	NR	Hyperactivity, Psychosis, aggressiveness	Alive
18	F	7	2	0.00 (10-34)	297 (68-188)	-	1	Global developmental delay and Irritability	NR	Irritability and Agitation	Alive
19	M	11	1	0.1 (10-34)	286 (67-124)	-	0.4	Dyspnea	NR	Austistic Behavior and Aggressiveness	Alive

NR: Not Reported. F: Female. M: Male.^a^Despite not having the result from NAGLU activity available, patient 12 was included because we had molecular analysis of NAGLU. NAGLU reference values vary according to method (Leucocytes 10-34 nmol/17h/mg; plasma 11-37 nmol/h/mL; filter paper>1.5 nmol/h/mL).

Characterization of developmental delay revealed the progressive nature of the disease (Table 2). Patients presented speech delay as the main early finding that physicians use to suspect MPS IIIB, and our data suggest a delay in forming 2-sillable words. Speech and language delay are reported as the most frequent initial symptoms of MPS IIIB, and hearing impairment, also observed in a significant fraction of our patients, may contribute to the speech and language delay (Shapiro et al., 2017). The mean reported age of onset of symptoms was 26 months (range: 0 - 72 months). Mean age of diagnosis was 7.2 years (n = 21, range 0.6-22 years). The mean value of NAGLU activity in leukocytes was 0.17± 0.16 nmol/17h/mg (range 0-0.5) (n=16). The average urinary GAG levels were 263 mg/mg creatinine (range 39-600 ug/mg creatinine) (n=17). MPS IIIB is a rare genetic disease, and, for this reason, there are no new updates regarding the panorama of variants found in the NAGLU gene. Part of the gene alterations present in our patient cohort are present in scarce reports in the literature. c.700C>T (p.Arg234Cys) pathogenic variant was previously reported by Ozkinay and colleagues (2021) in patients from Turkey, c.1811C>T (p.Pro604Leu) variant was previously reported by Ouesleti et al. (2011) in patients from Tunisia. The other variants were found primarily in patients from Brazil. According to previous work by the group (Montenegro et al., 2022), it was observed that the most frequent variant in the country (23%) is p.Leu496Pro. The other pathogenic variants presented in the present work are distributed heterogeneously throughout the regions of Brazil.

**Table 2 -:** Age of acquisition and loss of neuropsychomotor developmental milestones in Brazilian Muchopolyssacharidosis type IIIB patients.

Neuropsychomotor developmental milestone	Age	
	Mean	SD
Head Control		
Acquisition (Months) (n=4)	4.5	1.25
Loss (Years) (n=1)	7	-
Sitting without support		
Acquisition (Months) (n=9)	7.5	1.13
Loss	-	-
Walking		
Acquisition (Months) (n=8)	15.4	2.6
Loss (Years) (n=3)	7.7	2.1
Bladder sphincter control		
Acquisition (Months) (n=1)	30	-
Loss (years) (n=1)	18	-
Anal Sphincter Control		
Acquisition (Years) (n=1)	>3	-
Loss (years) (n=1)	18	-
Speaking two-syllable words		
Acquisition (Months) (n=8)	16	5.1
Loss (years) (n=4)	8	6.3
2-word Phrases		
Acquisition (months) (n=4)	30	11.9
Loss (years) (n=3)	9	7.8

In the following years, the patients started losing the ability of walk, followed by losing acquired speech and communicating skills, though high variability was observed, which may indicate that in our population we have patients with both slow and rapid progressive forms of the disease (Héron et al., 2011). Seizures are reported in 30% of MPS III patients (Héron *et al*., 2011), and are probably underestimated in our patients due to methodological reasons. The use of different antiepileptic drugs in this series suggests that it is indeed the case (Héron *et al*., 2011). 

MPS IIIB is mostly considered a neurologic disease, but our findings show that other somatic manifestations are also very prevalent. Among the most common somatic symptoms related to the onset of the disease are dysmorphic features (macrocephaly, gingival overgrowth and coarse facial features). These data corroborate the findings present in observations previously carried out by the group (Montenegro et al., 2022). 

As the disease progressed, it caught our attention that at least one cardiovascular abnormality was reported in almost 70% of our patients with available data, showing that these abnormalities are more common than expected. Figures 1 C  and D show the frequency of the main alterations observed. A recent study revealed that almost 40% of MPS IIIB patients develop valve disease, which was also the most common finding in our sample (Figure 1 c ) (Lin et al., 2019). Although usually mild to moderate, it is important to notice that one of our patients died due to cardiorespiratory arrest, which suggests that this aspect of the disease slowly progresses with age, and should be monitored carefully. 

Our data also point to a high frequency of other somatic abnormalities, including hepatosplenomegaly (74% of patients) and joint contractures (58%) (Table S1). Other findings such as dysostosis multiplex and even respiratory problems might be underrepresented and is a limitation of this work. As previously reported in a study (Berger et al., 2013), respiratory issues such as sleep apnea and upper airway infections were also present in our patients at high frequency (65%). Three of the patients died of pneumonia (15%), due to recurrent airway infections, and one had a sudden unexpected death during sleep. Most of the surgeries were performed in an attempt to ameliorate the respiratory function of the patients. Therefore, future therapies should focus not only on improving brain disease, but also on addressing the other somatic alterations, as they can lead to high morbidity and are often the cause of death for most patients (Whitley et al., 2018).

Our results corroborate previous reports and suggest that our Brazilian cohort of patients with MPS IIIB demonstrates to have mostly a severe disease phenotype (Valstar et al., 2011). Part of these phenotypic considerations must take into account the genotype of these patients (Montenegro et al., 2022), as well as the genetic profile of the Brazilian population, since a relationship like this has already been observed in studies with other types of MPS, such as Mucopolysaccharidosis type IVA (Santos-Lopes et al., 2021). Also, it might be that less severe forms of Sanfilippo are underdiagnosed.

Altogether, our results show diagnosis of MPS IIIB patients in the main medical centers from Brazil is performed with a small delay compared to developed countries. We also showed that MPS IIIB patients have abnormalities that progress with age. The disease progression was characterized in multiple systems, and hopefully these data will help designing appropriate future clinical trials and clinical management guidelines.

## Supplementary material

The following online material is available for this article:

Table S1 - Data from Brazilian MPS IIIB patients obtained at last recorded visit.
