# Supplementary material for: Disease progression in Sanfilippo type B: Case series of Brazilian patients
Source: Genet Mol Biol. 2024 Mar 8;47(1):e20230285. doi: 10.1590/1678-4685-GMB-2023-0285 (PMC10941728; doi:10.1590/1678-4685-GMB-2023-0285)
Supplement: Table S1 - [file 1415-4757-GMB-47-1-e20230285-s1.pdf]

Genetics and Molecular Biology
https://doi.org/10.1590/1678-4685-GMB-2023-0285

Supplementary Material to "Disease progression in Sanfilippo type B: Case series of Brazilian patients"

Table S1 - Data from Brazilian MPS IIIB patients obtained at last recorded visit.

No	WEIGHT (KG)	LENGHT (CM)	HEAD CIRCUMFERENCE (CM)	SLEEP APNEA/ABNORMALITIES	JOINT CONTRACTURES	HEPATOMEGALY	SPLENOMEGALY	HEARING ABNORMALITIES	OTHER FINDINGS
**1**	20.2	105	52	Yes	No	Yes	No	No	Thick hair, Downslanting palpebral fissures depressed nasal bridge , Gingival hyperplasia, Umbilical hernia
**2**	15.6	100	NR	Yes	No	Yes	No	NR	Dolicocephalia, depressed nose and retrognathism
**3**	12.1	84	48.5	Yes	No	No	No	No	Sinofrys, small nose with anteverted nostrils, gingival hyperplasia
**4**	32.8	152.5	NR	Yes	No	Yes	No	Yes	NR
**5**	16.5	98	54	Yes	No	Yes	Yes	No	Facial dysmorphism
**6**	49.6	151	58	NR	Yes	No	No	NR	Coarse face, thick eyebrows
**7**	32.3	130	58	Yes	Yes	No	No	NR	Hirsutism, thick eyebrows
**8**	11	88	50	Yes	Yes	Yes	Yes	NR	Bilateral inguinal hernia, Dysostosis multiplex, cortical atrophy
**9**	55	147	54	Yes	Yes	No	No	NR	NR
**10**	NR	NR	NR	No	NR	NR	NR	NR	Hirsutism

No	WEIGHT (KG)	LENGHT (CM)	HEAD CIRCUMFERENCE (CM)	SLEEP APNEA/ABNORMALITIES	JOINT CONTRACTURES	HEPATOMEGALY	SPLENOMEGALY	HEARING ABNORMALITIES	OTHER FINDINGS
**11**	NR	NR	NR	No	NR	NR	NR	NR	Ear infections
**12**	NR	NR	NR	NR	NR	NR	NR	Yes	Retinal deterioration at 18 years
**13**	NR	54	NR	NR	Yes	Yes	No	Yes	Coarse face, thick hair, cerebral atrophy
**14**	28	NR	56	No	Yes	Yes	Yes	NR	NR
**15**	32.2	NR	56	NR	Yes	No	No	NR	Spasticity, hyperreflexia, ventricular dilatation and hydrocephaly
**16**	27.9	120	55.5	Yes	No	Yes	No	No	NR
**17**	62	167	56	No	Yes	Yes	Yes	No	Tremors. Facial dyskinesia
**18**	23	113	52	NR	No	No	Yes	NR	NR
**19**	22.8	108	54	Yes	Yes	Yes	NR	No	NR
**Summary**	-	-	-	11 of 17	11 of 19	13 of 19	8 of 15	3 of 13	-

NR: Not Reported.
